# Detecting Mutually Exclusive Interactions in Protein-Protein Interaction Maps

**DOI:** 10.1371/journal.pone.0038765

**Published:** 2012-06-08

**Authors:** Carmen Sánchez Claros, Anna Tramontano

**Affiliations:** 1 Department of Physics, Sapienza University of Rome, Rome, Italy; 2 Institut Pasteur Fondazione Cenci-Bolognetti, Rome, Italy; 3 Center for Life Nano Science @Sapienza, Istituto Italiano di Tecnologia, Rome, Italy; Koç University, Turkey

## Abstract

Comprehensive protein interaction maps can complement genetic and biochemical experiments and allow the formulation of new hypotheses to be tested in the system of interest. The computational analysis of the maps may help to focus on interesting cases and thereby to appropriately prioritize the validation experiments. We show here that, by automatically comparing and analyzing structurally similar regions of proteins of known structure interacting with a common partner, it is possible to identify mutually exclusive interactions present in the maps with a sensitivity of 70% and a specificity higher than 85% and that, in about three fourth of the correctly identified complexes, we also correctly recognize at least one residue (five on average) belonging to the interaction interface. Given the present and continuously increasing number of proteins of known structure, the requirement of the knowledge of the structure of the interacting proteins does not substantially impact on the coverage of our strategy that can be estimated to be around 25%. We also introduce here the Estrella server that embodies this strategy, is designed for users interested in validating specific hypotheses about the functional role of a protein-protein interaction and it also allows access to pre-computed data for seven organisms.

## Introduction

The possibility of retrieving information about protein-protein interactions by high throughput experiments has led to the development of a number of methods for their analysis [1], [2]. Complementing the information provided by a protein-protein interaction (PPI) map with knowledge about the properties of the interacting proteins [3]–[5] is an effective route to exploit the power of high throughput data, add value to them and prioritize the subsequent validation experiments.

Here we address the problem of distinguishing whether, when more proteins interact with the same partner, they can do so simultaneously, i.e. whether their interaction is mutually exclusive, and show that this is possible by taking advantage of the continuously increasing information available on the three-dimensional structure of proteins. Most protein-protein interaction map studies have not considered the structural and chemical aspects of interactions; only a few authors have proposed to enrich protein networks with structural information of proteins [6], [7] for example by taking advantage of the structural similarity between the architecture of binding motifs in different proteins [8].

Here, we follow a rather different general strategy based on the hypothesis that, if two proteins interact with the same region of a common protein and therefore their interactions cannot be simultaneous, they might share a common surface region mediating the interaction.

To reliably identify these cases, we extract from a PPI all instances (hereafter called sub-networks) where at least three proteins of known structure interact with a common protein partner (the hub), compare their surface residues to identify structurally similar substructures comprising at least three residues using the FunClust public server [9] and list the results together with the level of structural and sequence similarity of the matching residues.

We applied this strategy to the interactomes of seven organisms. Using as a test set several (152) complexes of known structure included in the sub-networks, we show that Estrella allows the identification of mutually exclusive interactions with accuracy higher than 77%. The procedure also allows us to predict which residues are likely to be in the binding interface of the nodes, and in a significant number of cases (between 63% and 75%) we correctly identify at least one of them (5 on average) and this has obvious implications for helping to reduce the search space in docking procedures.

The percentage of sub-networks containing a sufficient number of proteins of known structure in the PPI maps, and therefore the coverage of the method, varies substantially for different organisms, as it could be expected, however it does reach 42% for human and more than 36% for yeast averaging at about 25%. (Table 1). These figures are bound to increase with time both thanks to the progress in experimental methods and, possibly, to the increasing reliability of modeling techniques [10]. For this reason, we also provide the method as an on-line tool (named Estrella) that can automatically perform the analysis on user provided datasets and permits access to the pre-computed results described here.

**Table 1:** Data used in the analysis and stored in the Estrella database.

Number of sub-networks containing:	HS	SC	DM	MM	CE	RN	EC	Total
All proteins	12294	6023	9570	3052	4934	838	695	37406
At least three node proteins of known structure.	5176	3971	156	13	65	78	171	9630
At least three non-redundant node proteins of known structure.	4598	3796	137	46	12	165	63	8817
Complexes of known structure involving the hub protein and least three node proteins.	81	62	0	1	0	1	7	152
Mutually exclusive interactions in complexes of known structure involving the hub protein and their node proteins.	64	59	0	1	0	1	3	128

HS: Homo sapiens, SC: Saccharomices cerevisiae, DM: Drosophila melanogaster, MM: Mus musculus, CE: Caenorhabditis elegans, RN: Rattus norvegicus, EC: Escherichia Coli.

The last two rows show the data used for validation.

## Results

### Method description

Given a PPI, the Estrella procedure consists in several steps. First the map is analyzed to retrieve sub-networks in which at least three proteins of known structure interact with a central protein. The structures of the proteins (identical sequences) are retrieved from the PDB database [11]. When more than one structure exists for a protein in the PDB, we selected the one with the best resolution and best coverage The surface residues of the entire proteins, including all their domains if any, are compared (all against all) using FunClust [9] to retrieve sets of structurally similar residues. The resulting sets are subsequently scored according to the level of conservation of the superimposed amino acids.

We analyzed the publicly available PPI maps for seven organisms [12] listed in Table 1 where we also show the percentage of sub-networks that include more than three proteins of known structure and can therefore be analyzed with our strategy. Both the pre-computed data for these organisms and the automatic pipeline for the analysis are publicly available at http://bl210.caspur.it/ESTRELLA/home.php.

In total, we could analyze 8817 sub-networks and in 7310 of them we could identify the presence of structurally similar regions in proteins interacting with the hub, which are candidates for being mutually exclusive interactions. For each sub-network we obtain several cluster, i.e. putative groups of three or more proteins sharing a structurally similar exposed region comprising at least three amino acids, and rank them according to the number of similar residues and the number of aligned pairs of structures.

### Validation

To test the effectiveness of the method, we extracted all sub-networks (152) where the experimentally determined structures of the complexes between the hub protein and more than one of the *bona fide* mutually exclusive interactors exists and used them to evaluate the effectiveness of the method.

Let us assume that there is a sub-network where a central protein interacts with M + N proteins where M are experimentally known to interact with overlapping regions of the central protein and N are not and that, for the same sub-network, Estrella produces a cluster of m proteins predicted to establish mutually exclusive interactions while n are not predicted to do so.

The True Positives (TP) are M 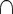 m; the False Positives are m 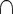 N; the True Negatives are N 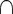 n and the False Negatives are n 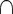 M. In other words, for each cluster, we count how many times we detect the correct mutually exclusive interactions (True Positives), how many times we include in the set of mutually exclusive proteins some that are not (False Positives), how many times we miss a mutually exclusive interaction (False Negatives) and, finally how many times we correctly predict that a protein of the sub-network does not bind to the same surface of the hub as the others in the sub-network.

**Figure 1:**
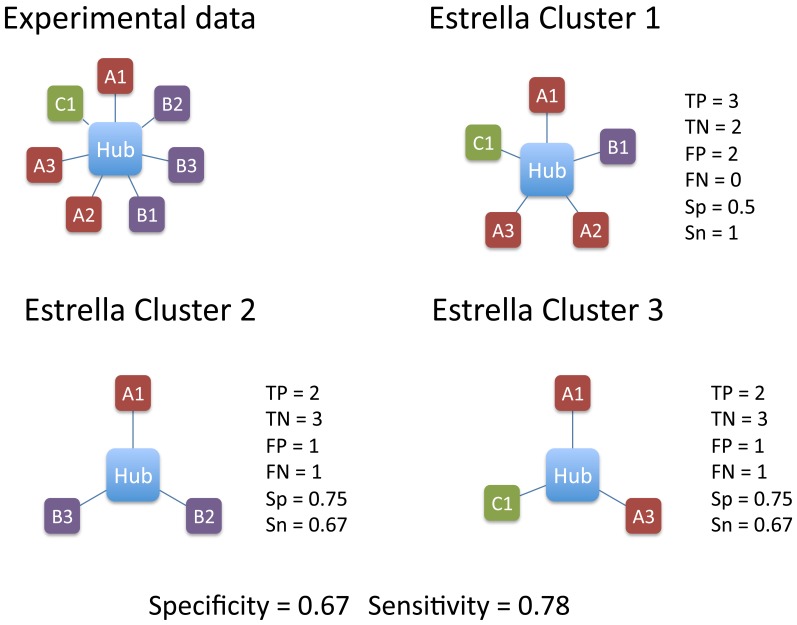
Exemplification of the way we compute the statistical parameters. In the left upper part of the figure we show the experimentally known situation where A1, A2 and A3 interact with the same region of the hub, the interaction of B1, B2 and B3 with the hub is also mutually exclusive, although they bind to a region different from that of the As. C1 binds to a region different from both the A and B binding sites. The example represents a possible set of sub-networks predicted as mutually exclusive by Estrella and the corresponding values for FP, TP, TN, FN, specificity (Sp) and sensitivity (Sn). The overall values for the specificity and sensitivity are computed as the average of the values for each identified cluster. In Cluster 1, the TP are A1, A2 and A3, the TN are B2 and B3, the FP are B1 and C1 and there are no FN. In Cluster 2, the TP are B2 and B3, the TN are A2, A3 and C1, the FP is A1 and the FN is B1. In Cluster 3, the TP are A1 and A3, the TN are B1, B2 and B3, the FP is C1 and the FN is A2. The overall values for the specificity and sensitivity are computed as the average of the values for each identified cluster.

Figure 1 schematizes the definition of these parameters in a more complex case.

As it can be appreciated from Table 2 and Table S1, the method has an average accuracy of about 77%, with a higher specificity (85%) than sensitivity (about 70%) when the results are averaged over all detected clusters. The sensitivity increases when only the first ranking cluster is considered at the expense of a 20% decrease in specificity. The overall accuracy is very similar in the two cases.

**Table 2:** Statistical parameters for the Estrella method applied to the sub-networks where the experimental structures of complexes between the hub protein and at least two partners are available.

	All clusters	First cluster
Correctly identified mutually exclusive node proteins (TP)	4428	260
Incorrectly identified mutually exclusive node proteins (FP)	878	95
Correctly identified non mutually exclusive node proteins (TN)	5162	57
Incorrectly identified non mutually exclusive node proteins (FN)	1898	36
Specificity = 100*TN/(TN+FP)	85.5	63
Sensitivity = 100*TP/(TP+FN)	70.0	88
Positive Predictive value = 100*TP/(TP+FP)	83.4	82
Negative Predictive Value = 100*TN/(TN+FN)	73.1	72
Accuracy = 100* (TP+TN)/(TP+FP+FN+TN)	77.6	79

Data are computed as the average of all clusters for each sub-network (first column) and only considering the first ranking clusters (second column).

Clearly, while the similarities that we detect indicate that the interactions can be mutually exclusive, we cannot exclude that other proteins binding to the same hub protein also cannot do so simultaneously since they could impair each other binding by steric hindrance.

It should also be mentioned (Table 3) that rarely we fail to identify more than one partner (less than 0.1% of the cases), while more often our prediction includes one protein that in reality does not establish a mutually exclusive interaction.

**Table 3:** Number of correctly identified interface residues in the correctly identified complexes.

	All clusters	First ranking cluster
Number of correctly predicted common interfaces complexes	1739	89
Total number of residues at the interface	34306	976
Number of correctly identified interface residues	9192	300
Number of common interfaces where at least one interface residue is correctly identified	1101	67

The identification of the common substructures often provides a correct prediction of the node binding sites as well. As shown in Table 3, we correctly identify 26% of the residues that are indeed buried in the complex interface on average. The figure raises to 31% if only the first ranking cluster is considered. Furthermore, we are able to correctly predict at least one interface residue in 63% of the cases (75% for the first ranking clusters) (Table 4). This is relevant, in our opinion, since the knowledge of which residues are likely to mediate an interaction can be used as a guide for docking algorithms to reduce the space that needs to be explored to identify the optimal interacting surfaces.

**Table 4:** Results of the Estrella procedure applied to sub-networks for which the experimental structure of the complexes is known.

Clusters	%
With more than one missing partner	8.72
With one missing partner	40.4
Perfectly defined	50.5
With one extra partner	0.23
With more than one extra partner	0.06

Data are shown for all clusters.

### Database and server

As mentioned above, the results for the seven analysed interactomes obtained from iRefIndex [12] are stored in the Estrella database available at http://bl210.caspur.it/ESTRELLA/home.php.

The database can be searched both with an organism and a protein name (using a number of database identifiers, see Methods) thus allowing the user to select a sub-network of interest, in which case she or he is directed to a page containing general information about the proteins in the sub-network and links to several other databases (CATH [13], PDB [14], UniProt [15], iRefIndex [12], SCOP [16], Genbank [17] and Gene Ontology [18]).

**Figure 2:**
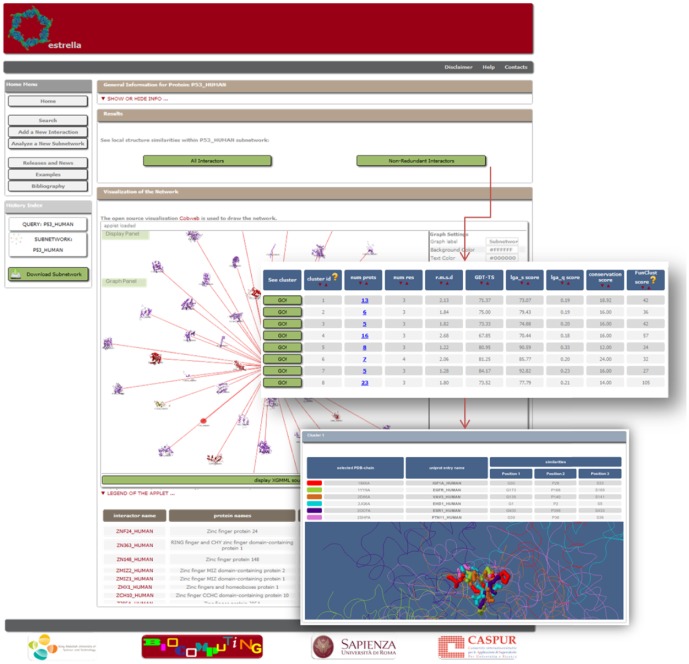
The output page of Estrella.

The sub-network is visualized in an interactive window [19] where nodes are proteins (those with a known structure are represented with a picture of their selected PDB chain) and edges are interactions. The display (and subsequent list of results) can be restricted to non-redundant proteins (defined as proteins sharing less than 30% sequence identity). The local structure similarity results are shown together with several information such as the RMSD between the residues identified in the putative common interface, the score obtained by the FunClust tool [9] (see Methods), the conservation of the interacting residues, the number of proteins and the GDT_TS value [20]. The FunClust score is used to rank the results, a Jmol applet [21] shows the best local structure superposition for the selected cluster and the results can be downloaded in tab-delimited format (Figure 2).

The complete automatic pipeline for generating the data can be accessed through Estrella. This permits to add a new interaction to an existing sub-network in the database, or to submit a completely new interaction sub-network. In these cases, the same analysis used to obtain the database is performed taking into account the new user-provided information (the results are not stored in the publicly available database). It is also possible to replace the coordinates for a protein included in the database, if a new structure becomes available.

## Discussion

The combination of the results of high throughput experiments and of their computational analysis is undoubtedly a powerful strategy for transforming the ever-growing amount of information that we are accumulating into knowledge.

Protein-protein interaction maps can be effectively used to transfer functional annotation from one protein to its interacting partners. The challenge consists in understanding at which level of granularity can the annotation be transferred, which also depends upon the mode in which the proteins interact. To this end, it is relevant to understand which interactions are mutually exclusive and which are the specific regions involved in the recognition process. To help solving these issues we developed and made available a system that we believe can effectively speed up the process of understanding the role of the gene products in a biological system.

In this paper we describe the implementation and the results of the strategy for identifying mutually exclusive interactions in a protein-protein interaction map based on the hypothesis that, if two or more proteins interact with the same region of a common partner protein, they might share similarity in their binding region.

We tested the idea using seven different interactomes from different organisms. The data are stored in a publicly available database, which we hope will be useful to life scientists. The method provides very satisfactorily results, especially since it has a rather high specificity (above 85%), thereby ensuring that scientists interested in a given biological process can retrieve essentially all of the *bona fide* mutually exclusive interactions in order to further validate the prediction. Equally important is, in our view, that only in a tiny fraction of the cases (less than 0.1%) the method incorrectly identifies more than one protein as part of a mutually exclusive interaction in a sub-network, and this implies that the number of necessary experiments to validate the results is greatly reduced.

When the simultaneous interaction of more than one protein with a common partner is correctly detected, the residues identified to be structurally similar among the nodes are very often the correct interface residues, and this implies that they can serve as constraints in docking experiments to reduce the search space.

Another observation that can be made from the results presented here is that the coverage of experimentally determined structures starts to be sufficient to allow their use in combination with different types of high throughput experiments.

We believe that the ever growing number of experimentally determined structures and of protein-protein interaction experiments, combined with the strategy presented here, that only requires the structure of the interaction partners and not of their complexes, also implemented in a completely automatic fashion and publicly accessible, is likely to add significant value to data produced in high-throughput experiments.

## Materials and Methods

Protein-protein interaction data were retrieved from iRefIndex release 7.0 (May 18th 2010) [12], a non-redundant and updated database, that provides an index of protein interactions available in several primary interaction databases, i.e. BIND [22], BioGRID [23], CORUM [24], DIP [25], HPRD [26], IntAct [27], MINT [28], MPact [29], MPPI [30] and OPHID [31]. Among the available interactomes, we selected those of the *Homo sapiens*, *Mus musculus*, *Rattus norvegicus*, *Drosophila melanogaster*, *Caenorabditis elegans*, *Escherichia coli* and *Saccharomyces cerevisiae*.

From these, we selected binary interactions with both partners annotated in the UniProt database [32] and those of known structure contained in the PDB database [14].

We define a sub-network as an interaction where a central protein is directly connected to at least three partners. To allow the user to select non-redundant interactions, we run PISCES [33] on our dataset and define as redundant those pairs of proteins sharing more than 30% sequence identity.

Solvent exposed residues were defined as those with more than 50% exposed surface with respect to the computed accessibility of the same residue type in an extended ALA-x-ALA tripeptide. Solvent accessibility was calculated using NACCESS using the isolated protein chains.

Structural similarity among solvent exposed residues of proteins interacting with the same hub was computed using FunClust [9] a publicly available tool that, given a set of protein structures, identifies structurally similar sets of residues within a predetermined threshold.

FunClust consists of a two-step procedure. In the first step, the Query3D algorithm (Ausiello, et al., 2005) identifies all the pairwise similarities among the chains within the sub-network. Query3D is a structure comparison method that searches for the largest subset of matching amino acids between two protein chains, regardless of whether hey are continuous in the chain, and only requiring them to be neighbors in space. It also takes into account their structural and biochemical similarity (according the PAM250 similarity matrix [34].

Residues are considered structurally similar when the RMSD of their C-alpha atom and of the geometric average of the side-chain atom coordinates is below 2.1 Å. The threshold for sequence similarity similarity according to the PAM250 matrix is 1.2. In the second step a clustering algorithm represents all the pairwise similarities as nodes of a graph, connecting them when the corresponding chains also share a group similarity, therefore identifying clusters of chains with a local structural similarity as connected paths in the graph. The clusters are sorted by an approximate significance score, called FunClust score, calculated by multiplying the number of residues in the group similarity by the number of chains belonging to the cluster [9]. In Estrella, the obtained sets are re-sorted after superposition using LGA [26] and the conservation score according to the BLOSUM 30 matrix [35].

The comparison between the predicted and experimental the results was performed using the PIA tool, included in PSAIA [36]. An interface is considered correctly predicted if Estrella identifies at least three residues that are part of the interface as defined by PIA.

The Estrella server is implemented using PHP and MySQL.

## Supporting Information

Table S1 **Results of the analysis of the seven interactomes listed in** **Table 1** **.** The table lists the SwissProt code of the hub protein, the PDB codes of the proteins forming the known complex and those identified by the Estrella procedure, the number of correctly identified and incorrectly identified residues. Data can be further analyzed by accessing the Estrella web server.
(XLS)
